# The association between uneven sex ratios and violence: Evidence from 6 Asian countries

**DOI:** 10.1371/journal.pone.0197516

**Published:** 2018-06-01

**Authors:** Nadia Diamond-Smith, Kara Rudolph

**Affiliations:** 1 Department of Epidemiology and Biostatistics and Global Health Sciences, University of California San Francisco, San Francisco, California, United States of America; 2 Department of Epidemiology, School of Public Health, University of California Berkeley, Berkeley, California, United States of America; University of Turku, FINLAND

## Abstract

It has been hypothesized that uneven sex ratios in the population could lead to increased violence. The objective of this analysis is to explore the relationship between uneven sex ratios in the population and violence. This analysis uses data collected from men in six Asian countries about their experiences and perpetration of violence. We combine this with region- and age specific sex ratios calculated from Census data to explore the relationship between sex ratios and violence using multilevel models. We find that men from region-age brackets with higher ratios of men to women are significantly more likely to report ever having raped a woman, having perpetrated intimate partner violence, or having used a weapon. We find no evidence for an association between sex ratios and reports of ever having raped a man.

## Background

In many Asian countries there are unequal numbers of men and women in the population, with more males than females [1]. Sex ratios in parts of Asia have increased from about parity in the 1980s to above 120 males for every 100 females in some countries by the 2000s, with India and China having some of the most uneven sex ratios [1]. This is thought to be founded on the long history of son preference in many parts of Asia, which, with increased access to sex-selective technology and abortion, has led to increasingly imbalanced sex ratios at birth [1]. Falling fertility in many countries in this region has further exacerbated the use of sex selective abortion, as families feel pressured to have sons within the first or second birth. After birth, discrimination against girls throughout infancy and childhood leads to excess mortality for girls, further imbalancing the sex ratio [2–4]. Thus, throughout much of the age pyramid, there are fewer women than men, although by older ages women still have higher life expectancy, even in countries like India with very imbalanced sex ratios at birth and through childhood [5].

It has been hypothesized that uneven sex ratios in the population might be associated with social instability and violence [6–8]. There are many potential mechanisms through which this could operate. With a shortage of women, it is more challenging for men to find marriage partners, for example, it has been estimated that there will be 40 million men remaining single in India and 32 million in China between 2020–2080 [8]. When there is a shortage of brides, it is likely that only the richest and most well-off men will be able to marry, leaving a large pool of men in lower socio-economic ranks without partners. Furthermore, since older men might be better off, they are more likely to be able to marry younger women, further exacerbating the shortage of brides for younger men.

Not being able to find a partner could increase competition and potentially violent competition through multiple pathways. First, it could increase violence between men, as men have to compete for wives. It has been hypothesized that when groups of men, especially young, unmarried men, spend time together, social dynamics can lead to increased violence [7]. Second, it could increase violence against women. Where there are not enough partners, men might be more likely to buy or potentially force sex (rape), either on a woman or another man. Past literature has suggested that despair (due to any number of factors) is associated with violence, and it is likely that despair combined with competition could push men to use violence [9]. Even among the men who do marry, large age gaps between men and women due to a shortage of women and thus men having to wait longer to marry, put women at low status in the household, as they are often less educated than their husbands. Age gaps and young age at marriage for women have been associated with increased risk of intimate partner violence and rape within the household, although the data is mixed for the association with age gaps [10].

Numerous disciplines (sociology, anthropology, psychology) have proposed theories for understanding violence, and specifically violence against women or intimate partner violence [11]. Dobash and Dobash (1979), taking a feminist lens, argue that in patriarchal cultures, which are based in men having power over women, violence against women can be used as a means of men maintaining power and dominance over women [12]. Son preference, leading to uneven sex ratios, is a common characteristic of patriarchal cultures [13]. Thus, this provides another framework for hypothesizing that patriarchy acts through imbalanced sex ratios to lead to increased violence towards women. Other theories of violence focus on how limited access to resources can lead to increased violence more broadly, as violence can be used to attain more resources [11].

There is another body of evidence that posits that the reverse will happen, and there will be less violence in regions where there is a scarcity of women (or an excess of men) [14, 15]. This literature points out that competition for women does not necessarily mean violent competition (for example, men could instead work very hard to compete financially) and also that where there is a scarcity of women and women are more able to choose partners, they may in fact choose more caring, nurturing and less violent men (and thus, men may become less violent in this situation). This argument might not hold in societies where women do not choose their partners (as in much of Asia with arranged marriages). Additionally, there is evidence that, although married men are less likely to be violent than married men, where there is an excess of women, men are actually less likely to marry and marriages are more unstable. Thus, clearly, evidence and theory behind the relationship between sex ratios and violence are both mixed.

Violence can be a difficult outcome to collect data on, due to stigma, fear, or even different cultural understandings about what is expected, and thus what violence even includes. Most studies collect data from women on their experiences of violence. However, the UN Multi-Country Study on Men and Violence in Asia and the Pacific collected data in 2010–2013 from men in urban and rural areas of six countries (Bangladesh, Cambodia, China, Indonesia, Papua New Guinea, and Sri Lanka) on their experiences of violence and perpetration of violence against women and other men. This study found that men’s reports of perpetrating physical violence ranged from 11.5% in rural Indonesia to 69.1% in Papua New Guinea, with an average across regions of 32.9% [16]. Reports of ever perpetrating sexual violence (rape) ranged from 10.4% in urban Bangladesh to 59.1% in Papua New Guinea, with an average of 24.3% across regions. Men’s reports of perpetrating rape against another man ranged from 1.5% in Indonesia, to 7.6% in Papua New Guinea. Similarly, men reporting that they were raped by another man ranged from 2.8% in China to 6.6% in Papua New Guinea. Given that there are few studies that collect data from men about violence and perceptions of violence, these data provide a unique opportunity to explore this issue.

Focusing in on the countries sampled in this data set, past literature has found evidence of strong son preference in Bangladesh [3, 17], China [13] and Indonesia [18]; moderate or no son preference in Cambodia [19, 20]; and no evidence of son preference in Sri Lanka [21] or Papua New Guinea [22]. In fact, there is even some evidence of higher rates of daughter preference than son preference in Cambodia and Indonesia [19]. Son preference does not necessarily lead to uneven sex ratios, thus not all of these countries have country-level sex ratio imbalance. However, uneven sex ratios at the population level have been observed in China [8, 23] and there is some evidence of uneven sex ratios in some age groups in Cambodia as well [24]. Bangladesh had gender differentials in mortality (not due to sex selective abortion), leading to uneven sex ratios in the past, however, the gender gap in mortality has closed in recent years [25].

One major limitation with past studies that have aimed to explore the relationship between sex ratios and violence is that many rely on cross sectional data and/or small samples collected in one geographic area. In order to better understand the relationship, being able to compare trends over time or heterogeneity across regions would be ideal. Given that data about violence is rarely collected, especially longitudinally, and that when it is collected it is rarely done so in a consistent manner across regions, it is hard to achieve this goal. Furthermore, data on sex ratios is often limited, and most often information about child sex ratios or the sex ratio at birth is reported, rather than age-specific sex ratios, at a population level.

In the UN Multi-Country Study on Men and Violence in Asia and the Pacific, data were collected in each country in multiple regions, including at least one urban and one rural area. We were able to explore the extent to which variation in sex ratios across age-region brackets explained variation in individual-level violence outcomes. We used age- and region-specific sex ratios as the level-two exposure, because it more accurately reflects the likely population of partners available for an individual. It also has the practical advantages of resulting in more level-two units, thus improving estimation.

The aim of this analysis is to explore the association between region and age-specific sex ratios on men’s reports of violence again women, against men, and use of a weapon in 6 Asian countries. We hypothesize that sex ratios favoring men will be associated with higher rates of all forms of violence.

## Materials and methods

The UN Multi-Country Study on Men and Violence in Asia and the Pacific, collected data from men from 2010–2013 in six countries (Bangladesh (2 sites), Cambodia (5 sites), China (2 sites), Indonesia (3 sites), Papua New Guinea (3 sites), and Sri Lanka (4 sites)) on their experiences of violence and perpetration of violence against women and other men. Data were collected from a total of N = 10,178 men across all 19 sites. This study was funded by Partners for Prevention, a United Nations Development Programme, United Nations Population Fund (UNFPA), United Nations Entity for Gender Equality and the Empowerment of Women (UN Women and United Nations Volunteers (UNV) regional joint programme for gender-based violence prevention in Asia and the Pacific. This data is currently not publically available and all necessary permissions were received for the use of this data.

For this analysis, we look at men’s report of ever raping a woman (Yes/No), ever perpetrating physical or sexual violence against an intimate partner (Yes/No), ever being in a fight with a weapon (Yes/No), and ever being raped by a man (Yes/No). We control for education (primary/none vs. more than primary), marital status (married versus not married), employment (employed versus unemployed), and an ordinal score of the level of poverty and food insecurity ranging from 1 to 7. These covariates were included because previous literate has found them to be associated with violence in other analysis of these data[26]. We also control for a measure of gender equity norms via the Gender Equitable Men Scale (GEM) (dichotomized at 24, the mean score in our sample), which includes items such as the acceptability of beating a woman, the importance of having a son, etc. We also include an interaction term between education and the GEM score as it improved model fit.

We combine these data with age-specific sex ratios from the regions of each of these countries from which the data were collected. The most relevant census data publically available for each country was 2011 in Bangladesh (http://203.112.218.65:8008/Census.aspx?MenuKey=43), 2008 in Cambodia (http://celade.cepal.org/redkhm/census/khm2008/), 2010 in China (not publically availiable), 2010 in Indonesia (http://sp2010.bps.go.id/index.php/site/tabel?tid=336&wid=3300000000), 2011 in Papua New Guinea (http://actnowpng.org/sites/default/files/2011%20Census%20National%20Report.pdf), and 2011 in Sri Lanka (http://www.statistics.gov.lk/PopHouSat/CPH2011/index.php?fileName=Activities/TentativelistofPublications). Age-specific sex ratios (the number of men divided by the number of women) were calculated for the age groups 15–24, 25–34, and 35–49. These age groups encompass the age ranges of men interviewed in the UN Multi-country Study on Men and Violence in Asia and the Pacific. Age-specific sex ratios were used to better represent the possible pool of marriage partners available in a given region. Regional sex ratio data (county, province, etc., depending on the country) were used, even though in some cases data were collected from a single city within that region. This is appropriate because (a) the marriage market and potential pool of mates is likely to include a slightly larger geographical area and (b) census data were only available at this level for many countries. This resulted in a total of 57 age-region sex ratios (the level-2 exposure).

There were little missing data (≤ 3%) for each of the variables in our analysis with the exception of intimate partner violence (IPV). Consequently, for each outcome, we conducted analyses on those with non-missing data. Because 19% of participants were missing data on IPV, we also performed a weighted analysis for this outcome using nonresponse weights (i.e., the inverse probability of responding, conditional on covariates included on our models in addition to others).

For each model, we checked for influential observations or those with high leverage. We compared regression coefficients dropping any potentially influential observations and found that our results were unchanged. Thus, the final models include all observations.

We conducted a multi-level analysis in which 10,178 men (the level 1 unit) were clustered into 57 region-age groups (the level 2 unit), using the lme4 package in R [27]. Using a multi-level logistic model, each outcome of interest was regressed on potential confounding variables at the individual level and sex ratios at the region-age group level. This decomposes the variance in the outcome into a within region-age group component and a between region-age group component, thus guarding against ecological fallacy. We used R version 3.3.1 for all analyses.

## Results

### Demographics

The age distribution for participants was similar across countries with the possible exception of China, which was more skewed toward older respondents (Table 1). The smallest proportion of men was in the young (18–24 year group) in all countries. Unemployment was low in Bangladesh (3.5%), China (12%), Indonesia (13.5%) and Sri Lanka (9.9%), but slightly higher in Cambodia (25.9%) and Papua New Guinea (30.9%). The vast majority of men were educated above primary school in Sri Lanka (90.7%), followed by China (85.8%) and Indonesia (82.5%). Levels of education were lower in Bangladesh (59.6%), followed by Cambodia (49.2%) and Papua New Guinea (43.2%). The greatest proportion of men were married in China (85.5%), followed by Cambodia (71.9%), Indonesia (67.2), Papua New Guinea (64.1%), Bangladesh (63%) and Sri Lanka (54.3%). Few men scored low on the poverty/food insecurity measure in China (6%), Indonesia (6.3%) and Sri Lanka (8%), and more men in Cambodia (30.8%), Bangladesh (28.4%) and Papua New Guinea (20.3%). The GEM score, measuring gender equity, ranges from 10 to 40 with a higher score representing more equitable gender views. China had the highest GEM score at 27.92, followed by Sri Lanka at 25.22. All of the other countries had GEM scores around 22–23.

**Table 1:** Participant characteristics, sex ratios, and violence related outcomes, by country.

	Bangladesh	Cambodia	China*	Indonesia	PNG	Sri Lanka
Total Population	2,395	1,8812	998	2,576	864	1,532
Age group N (percent)						
18–24	654 (27.3)	467 (25.8)	127 (12.7)	672 (26.1)	234 (27.1)	520 (33.9)
25–34	781 (32.6)	691 (38.1)	296 (29.7)	834 (32.4)	289 (33.4)	507 (33.1)
35–49	960 (40.1)	654 (36.1)	575 (57.6)	1,070 (41.5)	341 (39.5)	505 (33.0)
Unemployed (vs. employed) N (percent)	85 (3.5)	470 (25.9(	120 (12)	347 (13.5)	276 (30.9)	152 (9.9)
Education (some secondary or higher vs. None/primary N (percent)	1,428 (59.6)	891 (49.2)	855 (85.8)	2,214 (82.5)	373 (43.2)	1,389 (90.7)
Married/co-habiting (vs. not) N (percent)	1,508 (63.0)	1.303 (71.9)	852 (8.5)	1,730 (67.2)	554 (64.1)	832 (54.3)
Low Poverty/food insecurity (vs. high score) N (percent)	679 (28.4)	558 (30.8)	60 (6)	162 (6.3)	175 (20.3)	123 (8)
GEM score mean (SD) (higher is more equitable)	22.24 (2.44)	22.65 (4.19)	27.92 (3.76)	23.18 (2.88)	22.70 (4.36)	25.22 (5.26)
Sex Ratio (M/F) Mean (range)	0.916 (0.72–1.11)	0.960 (0.819–1.04)	-	1.05 (0.95–1.182)	1.03 (0.968–1.092)	0.926 (0.843–1.01)
Any physical or sexual intimate partner violence N (percent)	875 (55.95)	470 (32.7)	493 (51.46)	937 (39.89)	586 (80.38)	373 (32.89)
Any rape of partner, non-partner or gang rape of a woman N (percent)	280 (11.69)	369 (20.36)	222 (22.24)	808 (31.89)	530 (62.35)	20 (14.51)
Any rape of a man, alone or in a group N (percent)	64 (2.70)	58 (3.27)	16 (1.65)	40 (1.58)	65 (7.65)	38 (2.65)
Raped by a man N (percent)	112 (4.71)	66 (3.73)	27 (2.80)	117 (4.64)	56 (6.59)	50 (3.50)
Ever been in a fight with a weapon N (percent)	125 (5.26)	170 (9.59)	80 (8.19)	407 (16.09)	260 (30.52)	166 (11.53)

*China sex ratio not available for publication.

### Violence against women

Each additional 0.1 point increase in sex ratios favoring men is associated with men being more likely to report ever having perpetrated intimate partner violence (IPV), physical or sexual, against their partner (OR = 1.59, 95% confidence interval 1.26–2.01) (Table 2). Being poorer/more food insecure and reporting more equitable views on the GEM scale are significantly associated with lower odds of reporting IPV, and being married is significantly associated with higher odds.

**Table 2:** Odds ratios from multi-level logistic regression models by violence outcome. Estimates with 95% Confidence intervals.

	IPV^1^	Raped a woman	Was raped by a man	Fight with a weapon
Sex ratio^2^	1.59 (1.26, 2.01)	1.80 (1.40, 2.30)	1.07 (0.86, 1.33)	1.39 (1.12, 1.72)
Poverty/food insecurity	0.85 (0.82, 0.88)	0.84 (0.81, 0.88)	0.75 (0.70, 0.81)	0.97 (0.93, 1.02)
Education (less than high school)	0.92 (0.82, 1.03)	0.77 (0.68, 0.87)	0.81 (0.64, 1.02)	0.85 (0.73, 0.96)
GEM score	0.93 (0.91, 0.95)	0.94 (0.92, 0.96)	NA^3^	0.99 (0.98, 1.01)
Unemployed	NA^3^	1.23 (1.06, 1.41)	1.23 (0.94, 1.61)	1.17 (0.99, 1.39)
Married	2.22 (1.95, 2.53)	2.24 (1.92, 2.61)	0.80 (0.62, 1.02)	0.78 (0.66, 0.93)
Education*GEM (Interaction)	1.02 (1.00, 1.04)	1.01 (0.98, 1.03)	NA^3^	NA^3^

^1^ Results reported using nonresponse weights. Unweighted results were similar (not shown but available upon request).

^2^ Results for a 0.1 point increase in sex ratio.

^3^ Covariate was excluded to allow for model convergence.

Each additional 0.1 point increase in sex ratios (favoring men) is associated with men being more likely to report ever having raped a women (partner, non-partner or gang rape) (OR = 1.80, 95% confidence interval 1.40–2.30). Being poorer/more food insecure, having less education, and reporting more equitable views on the GEM scale are significantly associated with lower odds of reporting having perpetrated rape, and being unemployed or married are significantly associated with higher odds.

### Violence against men

There is no significant association between an increase in the sex ratio and men reporting having been raped by a man (OR = 1.07, 95% CI 0.86–1.33). Being poorer/more food insecure is associated with lower odds of reporting having been raped by a man.

### Violence with a weapon

Each additional 0.1 point increase in sex ratios (favoring men) is associated with increased odds of men reporting having used a weapon (OR = 1.39, 95% CI 1.12–1.72). Having less education and not being married are significantly associated with lower odds of reporting having been in a fight with a weapon.

As a falsification test, we estimated the association between sex ratios and an outcome that we expected would be unaffected—use of the healthcare system. Our hypothesis was confirmed; the association with sex ratios was small and nonsignificant (linear fixed-effect coefficient: 0.04 (95% CI -0.26, 0.35)).

## Discussion

We find evidence that uneven sex ratios are associated with men reporting having perpetrated intimate partner violence. This finding corroborates recent research in India that found that women living in communities with a more uneven sex ratio (more men) are more likely to report physical abuse by a husband, even after controlling for other household factors [28]. Other research in India found that uneven sex ratios in young people (age 15–39) was associated with the perception of young women being harassed more [29]. Additionally, we find evidence that uneven sex ratios are associated with men reporting having raped a woman. Rape against women has been receiving increasing attention throughout the world, but especially in South Asia and India, after the wide publicity of the fatal gang rape of a young woman in New Delhi in 2012 [30]. Reports of rapes of women in India have been increasing in the last few years, though it is unclear whether rape is actually increasing or the reporting of rapes is increasing [30]. Data from China has also found that women living in areas with a more imbalanced sex ratio were more likely to report being forced to have sex [31]. Our findings corroborate this previous research, suggesting that increasingly imbalanced sex ratios may be contributing to rape of women. We did not find evidence of an association between uneven sex ratios and rape of men. There is very little previous research on this topic, although a relationship has been hypothesized[32].

Our results also indicate that uneven sex ratios are associated with use of a weapon, which could be a proxy for community-level violence more broadly. Other scholars have postulated that uneven sex ratios will increase violence in general, not only against women. A study from China suggested that a more imbalanced sex ratio (by 0.01) was associated with increases in violent and property crimes by 5–6% These authors also postulate that men who are unmarried are more likely to join military groups, which could lead to increases in domestic or regional violence [33]. Another study explored the relationship between changes in sex ratios and violent and property crime in China between 1988–2004 [34]. They found that increasing imbalance in the sex ratio of people aged 16–25 is responsible for about one-seventh of the overall increase in these types of crime in China during that time period. Research in India has also found a relationship between sex ratios, violence, and homicide rates (as a whole, not only against women) [35]. Other research from India found that uneven sex ratios in younger populations (ages 15–39) were associated with people reporting more victimization by theft, breaking and entering, and assault [29]. Research in Mexico, which has high levels of male outmigration and high levels of regional violence due to the drug wars, has also found a relationship between sex ratios and homicide rates [36]. Specifically, using natural disasters as an instrumental variable that increases out migration and thus reduces the number of men compared to women, the authors found that 1 additional male per 100 females is associated with an increase in homicide rate of 0.4–0.6/100,000 people.

There are several limitations to this study. First, it is likely that there is an age gap between men and women in partnerships, so comparing men and women of the same age group to each other to calculate the sex ratio might not best represent the marriage market. However, since fertility is still rising in some of these countries, the sex ratios comparing different age groups would be imbalanced due to larger populations in the lower age cohorts. Second, the date of data collection of the UN study is not aligned exactly with the censuses from each country. While the Census years vary, all were within the time range that data collection occurred, with the exception of the Cambodia Census in 2008. It is unlikely that the age structure of the population changed significantly between 2008 and the time of the UN data collection for Cambodia (2010–2013). Furthermore, we were unable to incorporate population data on the exact cities or districts that were sampled in UN study. While the sex ratios might differ slightly using data from these specific geographies, it is also possible that the larger population size better captures the potential marriage market where people might search for partners outside of their immediate surroundings.

One of the main strengths of this analysis is that it uses data collected from men about their own experiences and perpetration of violence and other behaviors; however, this is also a limitation. Men may be likely to underreport all of the behaviors analyzed in this paper, given stigma against violence. Such underreporting would lead to underestimation of the effects in our analysis, and thus it is possible that the true magnitudes of associations are stronger than found here. However, we know of no evidence suggesting that living in a region with high sex ratios affects the likelihood of self-reporting an illegal behavior, such as these forms of violence. On the other hand, it is also possible that some of these behaviors could be over reported. In regions where there is a greater acceptability or positive norms related to violence (perhaps it being a sign of manliness), men may be more likely to report these behaviors. It should also be noted that there may be differences in under or over reporting of these behaviors between (and perhaps within) regions.

Given the complex and deeply cultural nature of the relationship between sex ratios and violence and the lack of an experiment or quasi-experiment intervening on sex ratios, establishing causality is not realistic. We hypothesize that shortages of women lead to increased violence against women and use of a weapon. It is also possible that living in a place where there is greater acceptance of violence against women and violence more broadly leads to son preference and thus to more uneven sex ratios. For example, parents living in a region where violence against women is common may be less likely to want to have a daughter, because she would be at additional risk of experiencing violence. Or, if there is an uneven sex ratio and men must compete more intensely to reproduce, they may use violence against each other and against women to do so. If violence is advantageous, then the reproducing pairs will include more violent men to secure an advantage by making more boys than girls. Most likely these two phenomena build on each other, and this two-way causality perpetuates equilibrium. Longitudinal data could possibly help understand this this two-way causality, specifically, how changes in sex ratios and changes violence against women influence each other over time. Overall, our analysis does not support the hypothesis that excess of men will lead to less violent men and less violence in general [14, 15]. This could be because, as mentioned above, even when there is an excess of men, women in these countries still do not have a large role in choosing their mates, and therefore cannot select for specific types of males (potentially non-violent).

Most of the limited research on the causes of uneven sex ratios has been in India and China due to the more extremely skewed sex ratios in those two countries. Our analysis generalizes that knowledge to other regions where the sex ratio imbalance is not as dramatic and son preference may or may not exist. We show that uneven sex ratios are associated with three different types of violence (rape, intimate partner violence, and use of weapons). This suggests that son preference and other causes of shortages of women can have societal impacts in many regions of the world, not just those that currently receive the most attention. Sex ratios have been becoming more unbalanced in many regions in the world, with an estimated peak of surplus women in 2035, before a hypothesized decline back to equity (some of the areas with the most uneven sex ratios have already begun to show signs of improvement) [23, 37]. If sex ratios become more imbalanced in the years to come due to son preference and sex selective abortion, discrimination against girls and women throughout their lives, or even potentially other factors such as migration, then it is possible that we will see increases in violence and unrest.
